# Density of TMEM119-positive microglial cells in postmortem cerebrospinal fluid as a surrogate marker for assessing complex neuropathological processes in the CNS

**DOI:** 10.1007/s00414-022-02863-5

**Published:** 2022-07-12

**Authors:** Simone Bohnert, Stefanie Trella, Ulrich Preiß, Helmut Heinsen, Michael Bohnert, Johann Zwirner, Marie-Ève Tremblay, Camelia-Maria Monoranu, Benjamin Ondruschka

**Affiliations:** 1 Institute of Forensic Medicine, University of Wuerzburg, Versbacher Str. 3, 97078 Wuerzburg, Germany; 2 Institute of Legal Medicine, University Medical Center Hamburg-Eppendorf, Butenfeld 34, 22529 Hamburg, Germany; 3 Department of Oral Sciences, University of Otago, 310 Great King Street, Dunedin, 9016 New Zealand; 4 Division of Medical Sciences, University of Victoria, Medical Sciences Building, Victoria, BC V8P5C2 Canada; 5 Department of Neuropathology, Institute of Pathology, University of Wuerzburg, Josef-Schneider Str. 2, 97080 Wuerzburg, Germany

**Keywords:** Cerebrospinal fluid, Forensic neuropathology, Forensic neurotraumatology, Immunohistochemistry, Immunocytochemistry, Biomarker

## Abstract

Routine coronal paraffin-sections through the dorsal frontal and parieto-occipital cortex of a total of sixty cases with divergent causes of death were immunohistochemically (IHC) stained with an antibody against TMEM119. Samples of cerebrospinal fluid (CSF) of the same cases were collected by suboccipital needle-puncture, subjected to centrifugation and processed as cytospin preparations stained with TMEM119. Both, cytospin preparations and sections were subjected to computer-assisted density measurements. The density of microglial TMEM119-positive cortical profiles correlated with that of cytospin results and with the density of TMEM119-positive microglial profiles in the medullary layer. There was no statistically significant correlation between the density of medullary TMEM119-positive profiles and the cytospin data. Cortical microglial cells were primarily encountered in supragranular layers I, II, and IIIa and in infragranular layers V and VI, the region of U-fibers and in circumscribed foci or spread in a diffuse manner and high density over the white matter. We have evidence that cortical microglia directly migrate into CSF without using the glympathic pathway. Microglia in the medullary layer shows a strong affinity to the adventitia of deep vessels in the myelin layer. Selected rapidly fatal cases including myocardial infarcts and drowning let us conclude that microglia in cortex and myelin layer can react rapidly and its reaction and migration is subject to pre-existing external and internal factors. Cytospin preparations proved to be a simple tool to analyze and assess complex changes in the CNS after rapid fatal damage. There is no statistically significant correlation between cytospin and postmortem interval. Therefore, the quantitative analyses of postmortem cytospins obviously reflect the neuropathology of the complete central nervous system. Cytospins provide forensic pathologists a rather simple and easy to perform method for the global assessment of CNS affliction.

## Introduction

Traumatic brain injury (TBI), vascular, infectious, neoplastic, and neurodegenerative diseases impact blood-brain-barrier (BBB) and cerebrospinal fluid (CSF)-brain-barrier (CSFB). These barriers become leaky, and, as a consequence, larger peptides, microbial metabolic substances, cytoskeletal, and intracellular neuronal and glial elements can be identified in the CSF [1, 2]. Since the CSF is permanently resorbed, interstitial fluid (ISF) influx carrying degenerated proteins and waste products from all parts of the central nervous system (CNS) replenish resorbed CSF [3]. Cellular, microbial, metabolic, and structural proteins are considered as biomarkers that act as surrogate markers of CNS neuropathology, e.g., after traumatization [4–15]. Research and diagnosis of enhanced CSF-cell density after inflammatory and tumor-related brain affliction have a long history [16–18]. Analysis of CSF cellular type and density estimations were so far not in the focus of forensic neuropathological diagnostic methods and received only marginal attention in forensic practice [19]. This is surprising since the CSF is anatomically well-protected by the skull and the spinal canal. Therefore, its topography predestines this body fluid to preserve the integrity of whole cells [20].

In addition to astrocytes, microglia, the resident macrophages of the CNS and the first cellular defense line whenever damage occurs (regardless if traumatic or not), play a critical and strategic role in mediating CNS inflammation [21, 22]. In 2016, TMEM119, a trans-membranous molecule [23], was proved to be a specific and robust microglial marker. Since that time, even though it can be downregulated in some contexts [24], it has served for the immunohistochemical demonstration of microglia response to fatal TBI [25] or intoxications [26]. In addition, modified histochemical protocols could detect microglia in postmortem CSF samples of cases with TBI [13].

In the present study, we have analyzed the quantitative correlation between the cortical and subcortical density of TMEM119-positive microglial profiles in paraffin sections with the density of TMEM119-positive microglial cells in cytospin preparations of the CSF after different fatalities and postmortem intervals (PMI). The objective of our study was to establish a protocol monitoring CSF density of TMEM119-positive microglial cells as a surrogate marker for the assessment of complex neuropathological processes in the CNS.

## Material and methods

### Sampling and processing

CSF was collected by semi-sterile puncture of the suboccipital space prior to opening of the skull and removal of the brain in the course of forensic autopsies at the Institute of Forensic Medicine, University of Wuerzburg. Unknown, non-natural and violent causes of death were the reason for forensic postmortem investigations. Small routine diagnostic tissue samples for histopathologic diagnoses were cut out from unfixed coronal sections through the dorsomedial frontal and parieto-occipital lobes. Qualitative and quantitative data from the tissue samples consisting of cortical gyri and adherent parts of the white matter were compared with corresponding data of cytospin preparations. Our study included 60 cases, 27 females and 33 males ranging in age between 24 and 97 years. Anamnestic data and PMI (from 10 to 713 h) are summarized in Table 1). This research study has been approved by the ethics committee of the University of Würzburg (local number 203/15).

**Table 1:** Characteristics of all cases of this study

**Case number** **and** ***CSF categorization***	**Sex**	**Age (years)**	**PMI (h)**	**Cause of death**	**Cardiopulmonary resuscitation (CPR)**	**Brain weight (g)**
***Strongly positive***						
1	F	59	58	Hypoxia	Yes	1330
2	F	84	227	Multiorgan failure	No	1265
3	M	61	57	Autoptically not determinable	Yes	1260
4	M	50	42	Cardiovascular failure	Yes	1320
5	F	86	70	Cardiovascular failure	Yes	1300
6	F	28	10	Hypoxia	Yes	1605
7	M	73	95	Traumatic brain injury	Yes	1400
8	M	68	34	Cardiovascular failure	No	1250
9	M	85	117	Cardiovascular failure	No	1535
10	M	47	105	Autoptically not determinable	Yes	1425
11	F	74	208	Isolated torso trauma	Yes	1265
12	M	24	142	Autoptically not determinable	No	1500
13	F	72	106	Hypoxia	Yes	1215
14	F	48	83	Multiorgan failure	Unknown	1390
15	F	66	294	Multiorgan failure	Unknown	1270
***Positive***						
1	F	81	153	Cardiovascular failure	No	1280
2	M	55	90	Hypoxia	Yes	1450
3	M	33	143	Autoptically not determinable	No	1220
4	M	89	312	Hypoxia	No	1575
5	M	50	105	Cardiovascular failure	No	1380
6	M	59	72	Intoxication by oxycodone, clomethiazole, ethanol	No	1405
7	M	60	136	Bleeding by polytrauma	Yes	1530
8	M	82	135	Cardiovascular failure	Yes	1420
9	F	58	93	Cardiovascular failure	Yes	1610
10	F	77	713	Multiorgan failure	Unknown	1480
11	M	57	466	Cardiovascular failure	No	1820
12	F	27	35	Hypoxia	Yes	1570
13	F	87	71	Multiorgan failure	Unknown	1240
14	M	80	184	Hypoxia	No	1530
15	M	55	122	Cardiovascular failure	No	1350
***Weak to negative***						
1	M	33	59	Cardioascular failure	Yes	1640
2	F	86	132	Cardiovascular failure	No	1180
3	M	85	150	Multiorgan failure	No	1405
4	F	80	75	Hypoxia	No	1370
5	M	39	120	Intoxication by codeine, methadone, diazepam	No	1490
6	F	95	98	Cardiovascular failure	No	1050
7	M	73	157	Gastrointestinal bleeding	No	1130
8	F	42	269	Intoxication by olanzapine	Yes	1230
9	M	37	105	Autoptically not determinable	Yes	1470
10	M	59	68	Autoptically not determinable	Yes	1565
11	F	35	165	Intoxication by diphenhydramine, ethanol	No	1245
12	F	89	89	Cardiovascular failure	No	1280
13	M	88	93	Traumatic brain injury	No	1180
14	F	63	55	Bleeding by ruptured aortic aneurysm	Yes	1430
15	F	78	81	Cardiovascular failure	No	1000
***Negative***						
1	M	30	130	Traumatic brain injury	No	1550
2	F	48	106	Autoptically not determinable	No	1190
3	F	97	216	Hypoxia	No	1165
4	F	76	105	Multiorgan failure	Unknown	1365
5	F	49	119	Cardiovascular failure	No	1420
6	F	80	123	Traumatic brain injury	No	1235
7	F	91	125	Traumatic brain injury	No	1180
8	M	66	185	Traumatic brain injury	No	1380
9	M	82	36	Traumatic brain injury	No	1310
10	M	90	132	Cardiovascular failure	No	1265
11	M	55	96	Cardiovascular failure	No	2050
12	M	53	72	Hypoxia	No	1500
13	M	62	61	Cardiovascular failure	Yes	1510
14	M	91	45	Cardiovascular failure	No	1200
15	M	25	40	Intoxication by heroin, ethanol	No	1520

The dissected cortical gyri were fixed in buffered 4% formalin, dehydrated, and paraffin embedded. The paraffin blocks were cut with a sliding microtome at 6 µm. Consecutive sections were mounted on microscope slides and routinely stained by Mayer’s hemalaun-eosin (HE) and immunohistochemically processed with a commercially available antibody against TMEM119 in a dilution of 1:1000 (Sigma, St. Louis, USA) [25] as primary antibody. The MultiLink Streptavidin-Peroxidase-Kit (BioGenex, San Ramon, USA) was used as secondary antibody. Control slides were stained by omitting the primary antibodies to test for unspecific staining in all staining charges.

For macroscopic orientation, digital images of HE and TMEM119 stained sections were taken with a Canon MP-E65 macrolens mounted on a Canon EOS MKII single lens reflex camera at 1.5 fold magnification depicting the complete sections. High-resolution RAW-files were adjusted for maximal contrast and transformed into TIF-files.

Microscopic digital images of the immunostained sections were taken with a Leica digital camera DMC 5400 mounted on a Leica DM6 B microscope at a constant using × 100 magnification (both Leica Microsystems Corporation, Wetzlar, Germany).

Five randomized images per case and slide free from staining and cutting artefacts were taken to obtain representative samples for each case. The total area of the images comprised 5.8 mm^2^ (1.16 mm^2^ per single photograph). For quantitative evaluation of the sections, an image processing software (Leica LASX, Wetzlar, Germany) was used as described before [26]. Prior to digital image analysis, parameters of cell morphology (size of profiles and staining intensity) were defined for the TMEM119 antibody. These parameters were invariably used throughout the measurements.

The software automatically copied the data into an Excel-macro table (Microsoft Corporation, Redmond, USA. The number of IH-positive profiles (microglial perikarya + major processes emanating from the perikarya) per field of view was expressed as number of immuno-positive profiles per square millimeter or simply density per mm^2^.

CSF samples were immediately centrifuged at 5000 rpm for 5 min at 4 °C and cytospin preparations were stained with the antibody mentioned above using an adapted protocol for immunocytochemistry [13] and identical dilutions .

The CSF samples were analyzed in the same way as the tissue sections. A total of five images were obtained from each cytospin preparation using the same lens and identical total magnification. The five samples per cytospin were taken in a clockwise manner at 12 o’clock, 3 o’clock, 6 o’clock, and 9 o’clock together with the center of each circular cytospin preparation (Fig. 2a).

Prerequisites for inclusion of subsequent cases in 2018 and 2019 successful CSF-sampling by suboccipital puncture, the application of an immunocytochemical TMEM119-protocol (cytospin preparation), and the availability of formalin-fixed paraffin-embedded tissue from the prefrontal and parietooccipital cortex of brains after successful CSF retrieval. Our 6-µm-thick paraffin sections included different cortical areas with more or less typical lamination and neuronal cell densities in the six isocortical layers. An exact areal diagnosis was illusive in 6 µm HE-stained paraffin sections. The presence of numerous stacked granule and small pyramidal cells at the border of the supragranular layer IIIc and infragranular layer V facilitated the distinction of pre- and postcentral cortical tissue.

Based on the number of TMEM119-positive cells in CSF, four categories (groups) were defined:

Case group *strongly positive* when > 30 TMEM119-positive profiles were detected, case group *positive* when > 10–30 TMEM119-positive profiles, case group *weak* to *negative* when 1–9 TMEM119-positive profiles, and case group *negative* when 0 TMEM119-positive profiles were detected.

### Statistical analysis

Excel Version 16.15 (Microsoft Corporation) and GraphPad Prism software version 8 (GraphPad Software, La Jolla, USA) were used for statistical evaluation: the Shapiro-Wilk normality test was used to test the distribution of the samples, an ordinary one-way ANOVA was used for parametric data of samples in addition to the post hoc Tukey’s multiple comparisons test, but a Kruskal-Wallis test was calculated for nonparametric data followed by Dunn’s test to avoid repetitive testing failure. Spearman coefficients were reported for the correlations, respectively. Adjusted *p* values equal or below 0.05 were considered statistically significant.

## Results

**Fig. 1:**
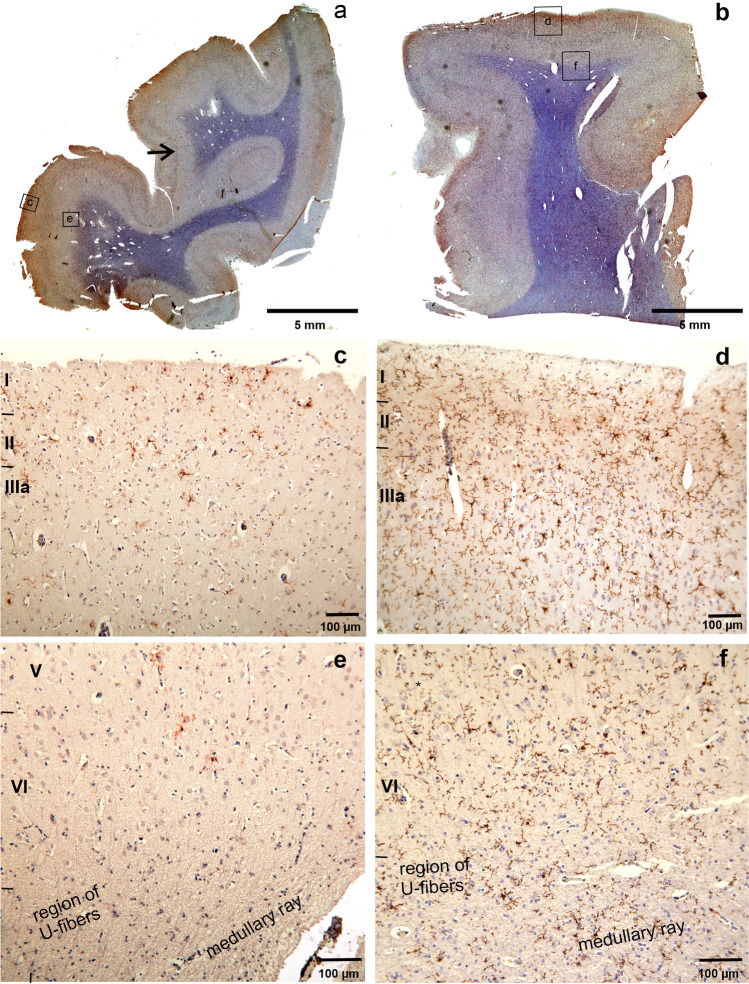
Low resolution overview of coronal sections through the isocortex of the parietal lobe (**a**, **b**). Female aged 91 years, TBI, PMI 125 h (**a**), and female aged 28 years, carbon monoxide intoxication, postmortem interval 10 h (**b**) In (**a**) IHC-staining is confined mainly to the crest of the gyri and weak or absent in the wall and fundus of sulci. Status cribrosus in the white matter of both gyri and perivascular loss of staining. Arrow points to prominent internal granular layer characteristic of cortical areae in parieto-occipital regions. Only few TMEM119-positive microglial cells with thin processes were seen in the supragranular layers I–III and in the deeper layers V–VI including U-fibers and the central medullary ray of gyri (**c**, **e**). Many processes of microglial cells appear blurred. This could be an effect of the long postmortem interval. Increase of TMEM119 microglial cells in the supragranular layer after intoxication in layers I–III (**d**) and infragranular layers V–VI (**f**) together with U-fibers and fibers coursing in the medullary ray of this gyrus. At the border between lamina II–I, microglial cells appear crowded and seem to loose their processes and microglial pericarya with few or without processes seem to head to the outer pia-covered region of layer I

Our observations and measurements were exclusively based on routine neuropathologic diagnoses of cases from our local forensic institute. It comprised a wide spectrum of lethal causes ranging from TBI to cardiovascular fatalities (CVF) (Table 1). High-resolution RAW or JPG files were indispensable to get an overview over the paraffin sections. The files could be magnified up to eight times without loss of details. At this magnification, intensely stained dot-like profiles proved to be single TMEM119-positive microglial cells. Their processes could not be recognized in the macroscopic overview. However, with microscopic lenses (× 100) their fine structure could be easily assessed (rectangles in Fig. 1a,b). In cases with lower cortical densities up to 100 profiles, TMEM119-positive microglial cells were not uniformly spread over the cortex but proved to be arranged in a more or less distinct laminar pattern (Fig. 1c-f). They clustered in the region of layer IIIa including the outer surface of layer II. In cytospin-TMEM119-negative cases the density of TMEM119-positive cortical microglial profiles was generally low. They appeared to be immersed in a diffusely stained neuropil (Fig. 1a,c). In cytospin-TMEM119-positive cases the density of cortical TMEM119-positive microglial cells increased and the neuropil became more intensely stained (Fig. 1b.d). In addition, TMEM119-positive profiles were also encountered in increasing numbers in layers IIIb, c and conspicuously in layers V and VI. The increased density of thin TMEM119-positive microglial processes most likely caused an increased diffuse neuropil staining in macroscopic overviews (Fig. 1e,f). U-fibers, a system of short-ranged association fibers, course between the lower border of layer VI and long-ranged projection- and association fibers comprising the central medullary ray. The layer of U-fibers together with layer VI and deep layer V proved to be a second hotspot of enhanced microglial density (Fig. 1f). TMEM119-positive microglial profiles were not confined to the region of U-fibers. They were also encountered in the deeper parts of the central medullary ray of cortical gyri that were continuous with long-range fiber systems in the hemispheric semi-oval center. We saw widely distributed foci with low number, confluent foci with a higher number of glial profiles and, finally, TMEM119-positive microglia densely populating the complete medullary region (Fig. 1a,b).

**Fig. 2:**
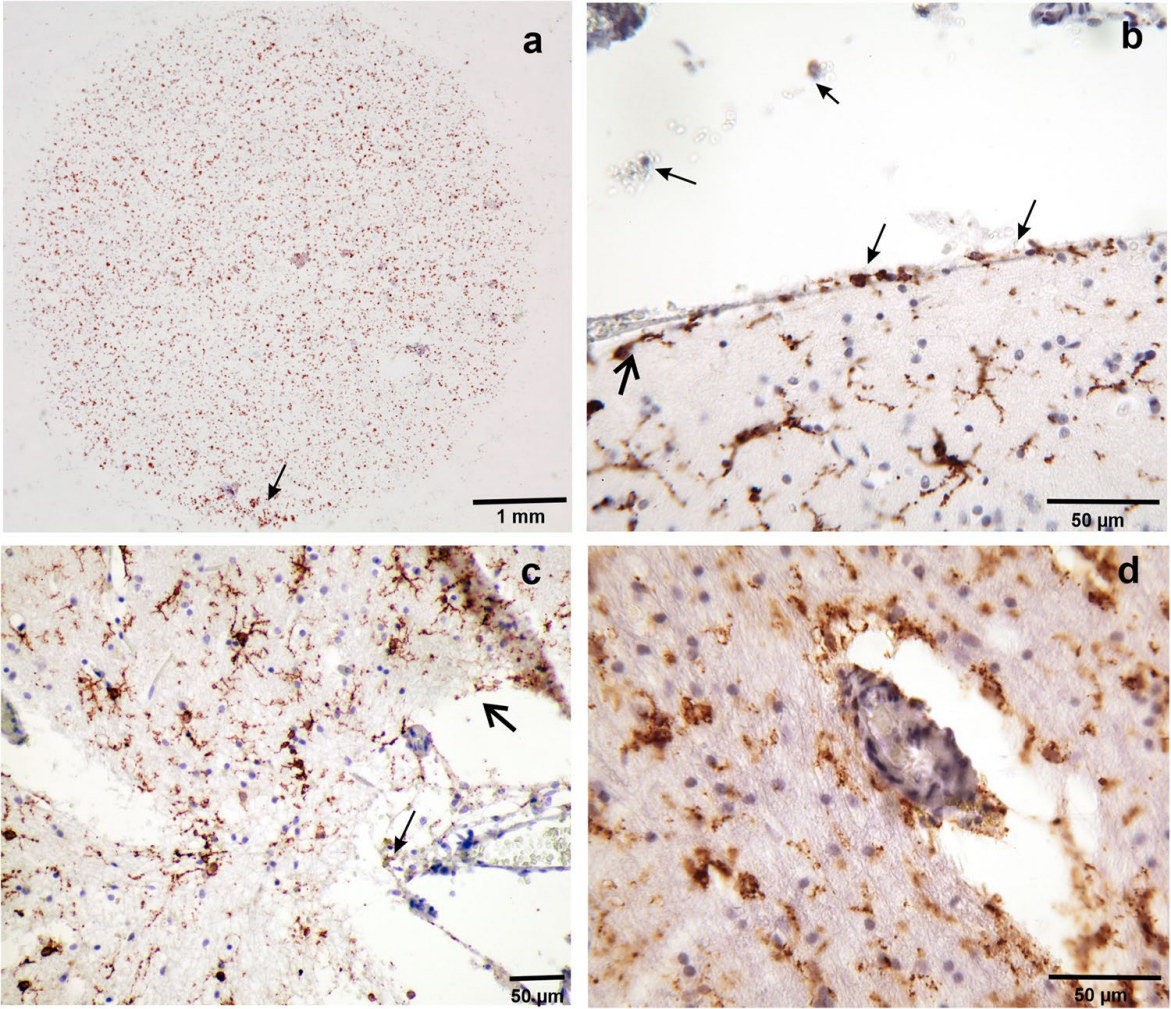
**a** Strongly positive cytospin preparation, female aged 48 years, sepsis, postmortem interval 83 h. Arrow points to grouped TMEM119-positive microglia (**b**) Same case as in **a**. The high-power microscopic image depicts microglial cells on their way to (big arrows) and through the pia (thin arrows) into the subarachnoid CSF. **c** Female aged 72 years, suffocation after aspiration, post-mortem-interval 106 h. Layer I at the fundus of a sulcus. The outer parts of the molecular layer appeared oedematous and groups of microglial cells and fragments of processes were interspersed in this spongy part of the molecular layer (thin arrow). The pia mater in this case is obviously detached and glia cells and fragments seemed to enter the CSF (thick arrow) but they were also attached to arachnoid fibers beyond the pia mater (thin arrow). **d** Same case as in **a**, subcortical myelin layer with tangentially cut vascular profile with TMEM119-positive profiles closely attached to the vascular adventitia. Shrinkage artifacts do not unequivocally disclose the mode of association or transition of numerous TMEM119-positive microglial cells from myelin layer to perivascular adventitia

TMEM119-positive microglia cells and the vascular system of the human central nervous system showed tissue-specific relationships. Profiles of bigger cortical arteries or veins were rarely visible in our paraffin-embedded tissue. However, venous and arterial profiles of different caliber were more frequently encountered in the medullary layer (Fig. 2c,d). Microglia and vessels of the medullary layer appeared to have a close relationship. Microglia distant from vessels were obviously intact, however, with decreasing distance to vessels microglial cells displayed fewer fine processes (similar to those microglial cells at layer I/II border) and were found nearby or within the perivascular space (Fig. 2d). Our paraffin sections were subject to considerable shrinkage and the relationship of perivascular TMEM119-positive profiles to the basal lamina could not be established with reasonable precision.

The topographical relationship of TMEM119-positive microglia to cortical vessels appeared to be less intimate (Fig. 2c). Vascular profiles were frequently encountered at the fundus of cortical gyri. At variance with vessels in the medullary layer, microglial cells rarely lost their processes to associate with the perivascular wall. We found far more vessel-independent single or groups of microglial cells with increasing density traversing the border of layer II with the deep parts of layer I. In this region, TMEM119-positive microglial cells seemed to divest their processes and dive into a spongious outer part of layer I (Fig. 2c thin arrow) or to align sub- and suprapially (Fig. 2b). Positive microglial profiles were also encountered in the arachnoidal space (Fig. 2b thin arrows) that in situ is flooded by CSF.

Our sample of a total of 60 cases comprised 27 female and 33 male cases with different causes of death and PMI. The effect of these parameters are depicted in a heatmap (Fig. 6). Brain-weight versus age correlated negatively and versus sex positively, cardiopulmonary resuscitation (CPR) versus PMI correlated negatively and versus CSF-intensity positively. There was no correlation between PMI and CSF-intensity and between CSF-intensity and brain weight.

**Fig. 3:**
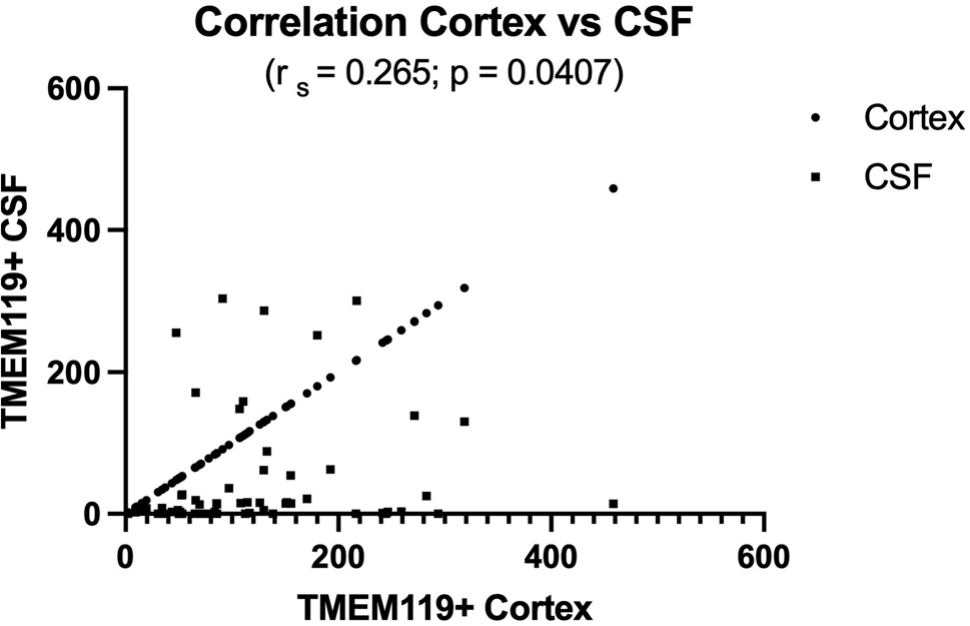
Correlation between cortical TMEM119-positive microglial density and CSF TMEM119-positive microglial density (cytospin preparations)

**Fig. 4:**
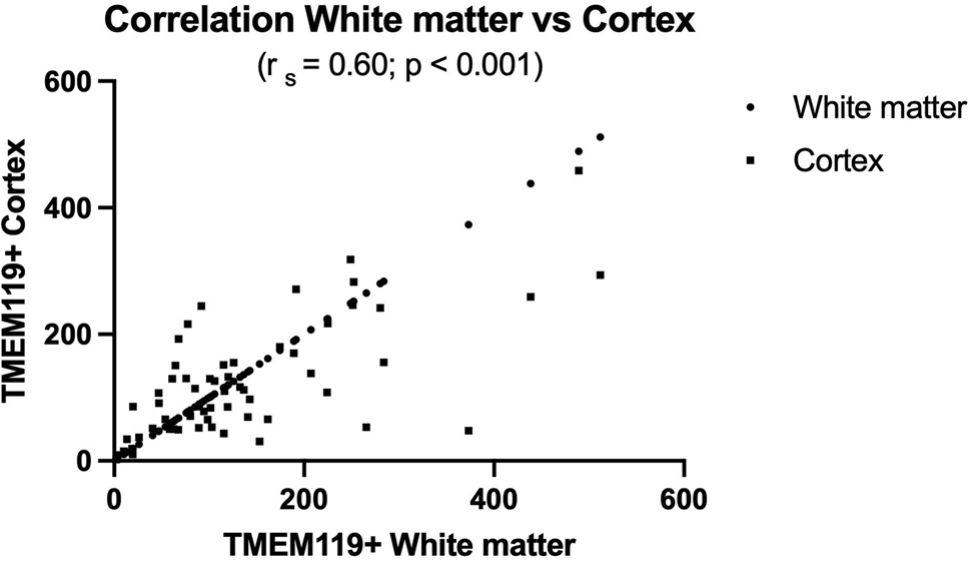
Correlation between cortical and white matter TMEM119-positive microglial density

**Fig. 5:**
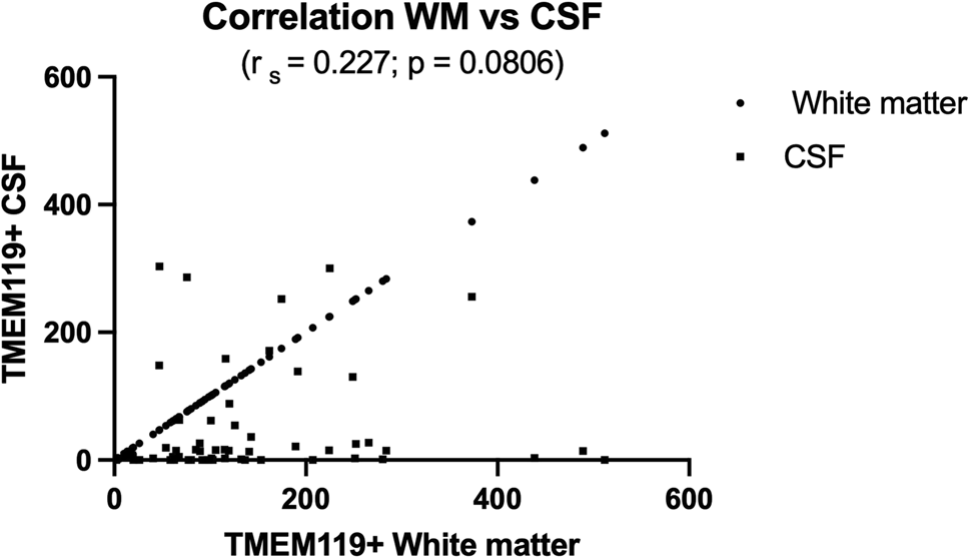
Correlation between white matter TMEM119-positive microglial cell density and CSF TMEM119-positive microglial density (cytospin preparations)

Our qualitative assessment of cortical and medullary TMEM119-positive microglial density was generally in line with computer assisted immuno-positive cell-density measurements in microscopic slides through cortex and cytospin preparations (Fig. 2a,b) This correlation proved to be statistically significant (Fig. 3). The high density of cortical TMEM119-positive microglial cells was accompanied with high density of microglial cells in the medullary layer (Fig. 1b,d,f). This impression was confirmed by the statistical analysis of cortex and white matter (Fig. 4). However, medullary layer microglial cell density did not show a statistically significant correlation with cytospin microglial measurements (Fig. 5).

Cortical glial density varied from 6 to 459, medullary layer density from 2 to 512, and cytospin density from 0 to 303. Correlating cortical TMEM119-positivity with causes of death yielded no uniform trend (unclear cause of death 459, acute heart failure with history of multiple older infarctions 318, drowning 294). Drowning was associated with the highest medullary layer TMEM119-positivity (512) in given cases in the absence of TMEM119-positive microglial perikarya in cytospin preparations. There were 11 cases with cortical TMEM119-positive profiles ranging from 216 to 459. In 6 of these cases cytospin-densities varied from 0 to 3, and in two cases with lethal myocardial infarction cytospin-positive preparations yielded 130 and 300 profiles. However, in these two cases the heart had experienced several preceding episodes of infarcts. TBI of the skull was associated with absent cytospin-profiles and 31 cortical profiles whereas after fracture of the 2nd vertebra we measured 256 TMEM119-positive profiles in the cytospin preparation and 48 cortical TMEM119-positive profiles.

## Discussion

**Fig. 6:**
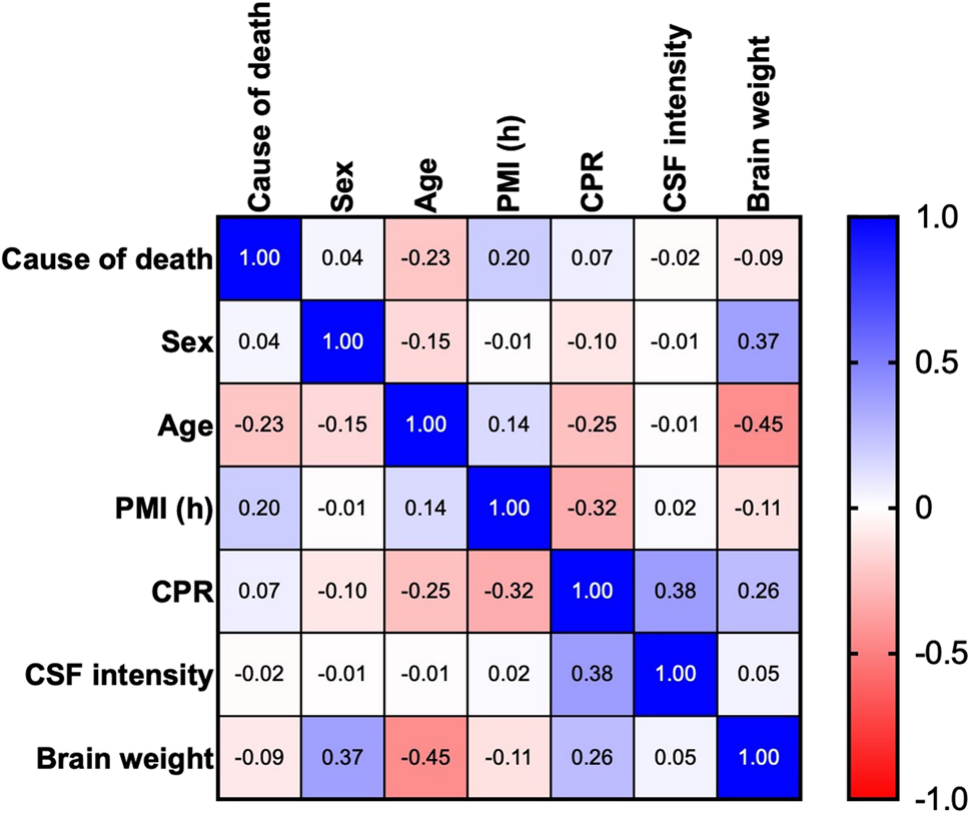
Heatmap. Correlations between cause of death, sex, age, postmortem interval (PMI), cardiopulmonal resuscitation (CPR), intensity of staining in CSF, and brain weight

Our sample contained a high number of cases that widely differed by age, causes of death, preexisting illness, and postmortem intervals. Male to female ratio was 27 females versus 33 male cases. Main confounding factors are depicted in a heatmap (Fig. 6) that proved the influence of age and sex on brain weight. In this aspect, our cases are in line with the majority of studies on these parameters [27, 28]. The negative correlation between CPR efforts and PMI implies the limitations of therapeutic CPR application. A victim of a traffic accident has a higher probability to be re-animated compared to a cadaver with initial signs of extended PMI or beginning autolysis. On the other hand, an extended PMI did not necessarily impact the staining intensity or cell density of CSF cytospins. In addition, the CSF density of TMEM-119-positive cells in our CPR-cases correlated positively. This could imply that CPR is able to mobilize transition of parenchymal TMEM119-positive microglia to CSF. It remains to be investigated to what extent and after what time intervals CPR intervention is able to mobilize microglia from the CNS. Nevertheless, the occipital subarachnoid space provides a long-term repository for the demonstration of TMEM119-positive microglia [20].

Brain tissue deterioration is subject to environmental factors and autolysis. Taken together, these confounding factors can have a massive impact on histological and immunohistochemical diagnosis and quantification of results. However, even cases with an extremely long PMI showed HE-stained neuronal and TMEM119-positive glial perikarya and their processes given the cadavers were quickly stored at 4 °C upon arrival. Cerebrovascular fatalities were always detrimental for CNS tissue quality and staining. Our IHC protocols were based on autoclaving paraffin sections glued on poly-l-lysine coated microscopic slides. In many cases, marked uneven staining was observed in the periphery of glued sections. This phenomenon indicates incipient peripheral floating off together with tears and missing parts. Another phenomenon was decreasing IHC staining of microglia from gyral crests, to walls and fundi with preserved HE staining of neuronal or glial nuclei and nucleoli irrespective of their topography. One explanation could be the effect of formalin fixation which is particularly marked in complete unsliced brains. In these scenarios we focused our quantitative analyses on well-stained regions of affected sections. Furthermore, our qualitative and quantitative analyses were confined to small regions of dorsomedial frontal and parieto-occipital lobes. These regions do not necessarily reflect the impact of circumscribed or extended lesions in other cerebral parts.

Our findings on fatalities with different causes most likely contributed to the considerable intra- and interindividual variations of the data depicted in Figs. 3, 4, and 5 and Table 1.

Cortical and medullary microglia can obviously react rapidly. Rapidly fatal myocardial infarctions were associated with absent or low density of TMEM119-positive profiles in cytospin preparations. The same was true for one case with drowning. On the other hand, two cases with lethal myorcardial infarction had an obvious history of multiple preceding infarcts. They were characterized by a high density of cortical and cytospin profiles. Local cortical or subcortical microglia can obviously react rapidly whereas CSF-transition of microglia is more time-consuming.

It is a matter of debate, how and when TMEM119-positive profiles originating in cerebral cortex and white matter gain access to the subarachnoidal and ventricular CSF. Migration of neuromelanin-loaded microglia targeting brainstem vessels and entering the cerebral circulation in Pick’s disease was already described by Scholz [29], and this original observation was extended and is presently summarized under the concept of the glymphatic pathway [30–37]. However, in this recent concept, migrating microglia is described to be enclosed by the basal lamina of meningeal arteries and/or veins and it is not debated when, where and how cortical or medullary TMEM119-positive microglia enter the CSF. We provide evidence that cortical TMEM119-positive microglia directly gain access to or are expelled into the CSF. Unfortunately, we had no periventricular white matter in our archived cases to show a migration of medullary TMEM119-positive microglia through intact ventricular ependyma or leakage through a defective one. The highly gyrated isocortex provides a far higher surface for resident microglial migration/leakage into the CSF compared with deep white matter microglia. This could explain the statistically significant contribution of cortical microglia to microglial cytospin positivity and the absence of a statistically significant contribution of white matter microglia to the latter in our cases. On the other hand, the significant quantitative correlation of white matter TMEM119 positive profiles to TMEM-positive cortical profiles obviously reflects the close functional relationship between cortex and white matter. In summary, postmortem sampling and quantitative assessment of TMEM119 IH-staining of CSF is a simple and reliable tool to diagnose the impact of different causes of death on the CNS in the course of forensic autopsies.

## Limitations

We have analyzed a relevant number of cases categorized by density of TMEM119-positive profiles in cytospin preparations and density measurements of microglial profiles in cortex and white matter. Our small-sized paraffin sections do not necessarily reflect the impact of more massively affected remote regions in our correlation studies. The same is true for differences in IHC-staining of gyral crest versus wall and fundi. We could not address the question of concomitant local and distant neuron loss and the role of remote antero- and retrograde degeneration as drivers in microglia reaction, proliferation, and recycling. In addition, the anamnestic data including PMI, biographical, and clinical data are vague and incomplete. Only TBIs and other sudden fatalities in the absence of preexisting diseases could elucidate different scenarios of glial reaction in cytospin preparations, cortex, and medullary layer. A correlative quantitative study on the extent and topography of TBI in the complete CNS of healthy young adults versus TMEM119-positive microglia in the CSF would be free of confounding diseases and could corroborate a straightforward relation between CNS lesion and local/CSF microglial reaction.

## Conclusion

Our observations on laminar distribution of microglia and its quantitative interactions with TMEM119-positive microglia in the CSF provide new insights into a fast-reacting highly dynamic system. Cytospin analyses reflect a versatile neurobio- and pathobiology of human microglia. They supplement the growing concept of glymphatic pathways and warrant further studies to clarify diverging quantitative differences in cases with similar causes of death together with the reason of selective laminar vulnerability at the border of isocortical layers I/II and deep layers V/VI including U-fibers. Forensic autopsy tissue proved invaluable in addressing fundamental neurobiologic and pathologic questions and interpretation of results of animal studies [38, 39].
